# A Cross-Sectional Study on the Prevalence, Risk Factors, and Impact of Myopia Among Medical Students in Andhra Pradesh, India

**DOI:** 10.7759/cureus.88760

**Published:** 2025-07-25

**Authors:** Sri Rama Chandra Murthy Manchikanty, John Shekhar Raju M., Usha C., Venkata Dileep Kumar Veldi, Keerthi Sanapala, Harleen Kaur, Tarakeswara Rao Attada, Anirudh Srinivas Teja Peela, Sai Nikhitha Amara, Singupuram Sai Teja, Anika Goel, Amogh Verma

**Affiliations:** 1 Ophthalmology, Gayatri Vidya Parishad Institute of Health Care and Medical Technology, Visakhapatnam, IND; 2 Ophthalmology, Maharajah’s Institute of Medical Sciences, Vizianagaram, IND; 3 Anatomy, Malla Reddy Institute of Medical Sciences, Suraram, IND; 4 Medicine, Gayatri Vidya Parishad Institute of Health Care and Medical Technology, Visakhapatnam, IND; 5 Medicine, Andhra Medical College, Visakhapatnam, IND; 6 Medicine, Government Medical College Patiala, Patiala, IND; 7 Medicine, NRI Institute of Medical Sciences, Visakhapatnam, IND; 8 Medicine, Kakatiya Medical College, Hyderabad, IND; 9 Medicine, Rama Medical College Hospital and Research Centre, Delhi, IND

**Keywords:** academic performance, eye care, medical students, myopia, near work, outdoor activity, prevalence, risk factors, screen time, vision impairment

## Abstract

Background: Myopia, or nearsightedness, is increasingly prevalent among students, largely due to prolonged screen time, near work (visually demanding activities performed at a short distance (typically less than 40 cm)), and reduced outdoor activity. Medical students, in particular, are at higher risk due to academic pressure and digital exposure, affecting their vision, performance, and well-being. Early detection and prevention are essential. This study investigates the prevalence, risk factors (genetics, near work, screen use, outdoor activity), and impact of myopia on daily life and academics to support awareness and better eye care practices.

Methodology: This cross-sectional study was conducted among undergraduate medical students in three tertiary care hospitals in Andhra Pradesh, India, over four months (August-December 2024). Using probability proportional to size sampling (PPSS), 1,506 students across different academic years were selected. Eligible participants were enrolled students who consented to participate, while those with hypermetropia or severe ocular conditions were excluded. Data were collected via a structured questionnaire covering demographics, myopia history, lifestyle habits, and academic performance, as well visual discomfort, psychological well-being, and myopia correction practices. Ethical approval was obtained, confidentiality was maintained, and pre-testing ensured data accuracy. Descriptive analysis determined myopia prevalence, and chi-squared tests were used to assess associations with genetics, screen time, sleep patterns, and academic performance.

Results: The overall prevalence of myopia among the medical students was 947 (61.55%), with significant associations between demographic and lifestyle factors. The highest prevalence was in the 20-22 age group (412, 62.24%) and the lowest in the 23-25 group (23, 48.94%), though the difference was not statistically significant (χ² = 5.23, p = 0.51). A total of 597 (65.19%) female students had a significantly higher prevalence than male students (322, 54.58%) (χ² = 17.38, p < 0.001). Myopia prevalence varied by academic year (χ² = 743.7, p < 0.001), peaking in third-year students (244, 70.11%) and lowest in fourth-year students (161, 46.55%). Family history was a strong predictor (χ² = 103.07, p < 0.001), with a higher prevalence among those with genetic predisposition (83.57%). Early onset (ages 5-10 years) was associated with greater severity (χ² = 248.03, p < 0.001), with 58 (51.33%) developing moderate and 25 (22.12%) high myopia. The associations with near work (χ² = 15.14, p = 0.08), screen time (χ² = 12.09, p = 0.2), and sleep patterns (χ² = 4.4, p = 0.88) were weak. Psychological distress was correlated with severity (χ² = 28.26, p < 0.05), and 50% of the high myopes reported severe limitations in daily activities.

Conclusion: This study highlights the high prevalence of myopia among medical students in Andhra Pradesh, linking it to lifestyle factors, such as prolonged near work, excessive screen time, and limited outdoor activity. Female students, particularly in their first year, were a higher risk. These findings emphasizes the importance of early detection through regular eye check-ups and lifestyle adjustments to slow myopia progression. Additionally, this study underscores the impact of myopia on academics, daily life, and mental well-being, reinforcing the need for targeted vision-care strategies and preventive measures in academic settings.

## Introduction

Myopia or nearsightedness is a refractive error that occurs when far away objects appear hazy while close ones are clearly visible. The prevalence of myopia is increasing globally and has become a significant public health concern. Estimates suggest that nearly 4.76 billion people may be affected by 2050, with nearly 10% expected to have high myopia, highlighting the growing burden worldwide. This phenomenon has been seen to correlate with a person’s lifestyle and environment, which includes possible factors such as excessive screen time, less time spent outside, and too much near work (visually demanding activities performed at a short distance (typically less than 40 cm)) being done [1]. Medical students belong to this category because of their excessive academic activities which puts them at a high risk of developing myopia [2].

Recent studies indicate that greater focus is placed on the need to identify the condition and intervene quickly to halt the increase in the severity of myopia. Zhou et al. further supported students ability to prevent myopia by exercising will power by refraining from excessive much screen time and changing to social events instead [3] . Zhou et al. and Zhang et al. stress the importance of diet and study intervals to reduce the chances of developing nearsightedness [3,4]. These are important factors that need to be addressed especially for medical students who require high visual abilities in reading and using a computer frequently.

In this regard, this study focuses on the problem of myopia among medical students, paying special attention to its prevalence, causes, and consequences. By examining the challenges associated with myopia in an academic setting, this research aimed to enhance awareness and promote effective eye care practices among students, contributing to a better understanding of myopia management and prevention.

Objectives

This study aimed to estimate the prevalence of myopia among medical students and examine associated risk factors, including genetic predisposition, near work, outdoor exposure, screen time, and sleep patterns. It also sought to assess the impact of myopia on academic performance and quality of life, focusing on visual discomfort, psychological well-being, daily activities, and myopia-correction practices.

## Materials and methods

Study design 

This study used an observational cross-sectional design to evaluate the prevalence, awareness, risk factors, and effect of myopia among undergraduate medical students in Andhra Pradesh, India. It was conducted in tertiary care hospitals in the state, with data gathered during the academic year.

Study population 

The study population comprised undergraduate medical students attending medical colleges working in tertiary care hospitals in the state of Andhra Pradesh in India over a period of four months, from August 2024 through December 2024, to conduct data collection at three different medical colleges across Andhra Pradesh.

Sample size 

The required sample size for estimating a prevalence of 70.3% was calculated to be 321 participants with 95% confidence level and a 5% margin of error. This estimate was based on a study conducted by Sandhu et al. [5]. However, a larger sample size of 1,564 was collected and 1,506 undergraduate medical students was selected based on inclusion criteria to enable subgroup analysis, improve accuracy, and reduce bias. To minimize selection bias, probability proportional to size sampling (PPSS) was employed to ensure representation across different academic years. Additionally, carefully defined inclusion and exclusion criteria helped maintain sample consistency, and trained data collectors ensured standardized data collection procedures. The total sample of 1,506 students was distributed among the three medical colleges, with each college contributing 502 students. The sample was stratified by academic year using the PPSS to ensure proportional representation, with an actual sample size of 600 students from the first year and 450 students each from the second, third, and fourth years. This resulted in 462 students (30.68%) from the first year, and 348 students (23.11%) each from the second, third, and fourth years. This larger, stratified sample facilitates a more detailed examination of variations in myopia prevalence and its associated factors across different academic years and demographic subgroups, thereby contributing to a more comprehensive understanding of the issue.

Inclusion and exclusion criteria 

Undergraduate medical students currently enrolled in medical colleges within the tertiary care hospital across Andhra Pradesh and students who consented to participate in the study were included. Students with hypermetropia and pre-existing severe ocular conditions other than myopia were excluded from the study.

Data collection procedures 

Data were collected via a structured questionnaire (see Appendix 1) covering demographics, myopia history, lifestyle habits, and academic performance. The questionnaire was administered physically by visiting the classrooms and instructing students on how to complete it. The study collected comprehensive data on medical students' demographics, including age, sex, and year of study, along with myopia history such as presence, age at diagnosis, and family history. Data were also collected on lifestyle factors such as near work hours and outdoor activities along with academic performance metrics. Quality of life aspects such as visual discomfort, psychological well-being, and daily activities were also taken into consideration. Furthermore, to provide a detailed understanding of myopia management among medical students, practices related to myopia correction, including the use of corrective lenses and adherence to treatment were explored. Myopia was graded based on spherical equivalent refractive error as low (-0.50 D to -3.00 D), moderate (-3.00 D to -6.00 D), and high (> -6.00 D), consistent with standard clinical definitions.

Quality control and confidentiality 

To ensure clarity and validity, the questionnaire was pre-tested with a small group of students. Data collectors were trained to administer the questionnaires and maintain confidentiality. The collected data were periodically reviewed to ensure completeness and accuracy. By anonymizing data and securely storing responses participant confidentiality was ensured.

Descriptive analysis and inferential statistics 

The prevalence of myopia among the sampled medical students was calculated, along with summarized demographic characteristics, grades of myopia, lifestyle factors, and academic performance metrics. Chi-squared tests were applied to assess the relationship between myopia grades and various factors, including genetic predisposition, near work hours, screen time, outdoor exposure, sleep patterns, academic performance, and quality of life measures such as visual discomfort, psychological well-being and daily activities.

Ethical considerations 

Ethical approval was obtained from the Institutional Review Board of the medical college of the tertiary care hospital. (RC. No: GVPIHCMT/IEC/20240725/12, dated July 25, 2024, IEC Reg. No.-EC/NEW/INST/2022/3026)

Informed consent and voluntary participation 

Participants were thoroughly informed about the study’s objectives, procedures, and rights, including the assurance of the confidentiality off their responses. All participants provided written informed consent prior to participation. In addition, participation was entirely voluntary, allowing students the freedom to withdraw from the study at any time without any consequences or penalties.

## Results

In our study, the overall prevalence of myopia among medical students was 947 (61.55%), highlighting the significant occurrence of the condition within this specific academic group. The analysis of myopia prevalence among the students revealed several significant associations between various demographic and lifestyle factors.

This study revealed varying prevalence rates of myopia across different age ranges, though statistical significance was not reached (χ² = 5.23, p = 0.51). The highest prevalence was observed in the 20-22 age group (412, 62.24%), followed by the 17-19 age group with a prevalence of 484 (60.73%). The 23-25 age group exhibited the lowest prevalence of 23 (48.94%). Sex was significantly correlated with myopia severity (χ² = 17.38, p < 0.001), indicating notable differences in prevalence and severity between sexes. Specifically, the prevalence of myopia was higher in women (597, 65.19%) vs in men (322, 54.58%). Additionally, female students exhibited a higher percentage of low myopia (364, 39.78%) compared to male students (189, 32.03%). This finding indicates that female students are more likely to develop moderate myopia than male students (Table 1).

**Table 1:** Demographic and academic characteristics of study participants

Variables	Category	Frequency	Percentage
Age range	17 to 19 years	797	52.92%
	20 to 22 years	662	43.96%
	23 to 25 years	47	3.12%
Gender	Female	916	60.82%
	Male	590	39.18%
Year of study	1st Year	462	30.68%
	2nd Year	348	23.11%
	3rd Year	348	23.11%
	4th Year	348	23.11%

Significant variations were observed in the prevalence of myopia across academic years (χ² = 743.7, p < 0.001). Prevalence rates were highest in the third year at 244 (70.11%) and lowest in the fourth year at 161 (46.55%). The first-year students exhibited a myopia prevalence of 317 (68.83%), which was relatively high, whereas the second-year students demonstrated a lower prevalence of 197 (56.61%) (Table 2). 

**Table 2:** Association between myopia severity and demographic factors Pearson's chi-squared test; p < 0.001: Highly significant; p < 0.05: Significant

Variable	Category	Emmetropia (n, %)	Low myopia (n, %)	Moderate myopia (n, %)	High myopia (n, %)	Total	Chi-squared value	p-value	Degree of freedom
Age range	17 to 19 years	313 (39.22%)	287 (36.02%)	173 (21.72%)	24 (3.01%)	797	5.23	0.51	6
	20 to 22 years	250 (37.73%)	250 (37.73%)	142 (21.45%)	20 (3.02%)	662			
	23 to 25 years	24 (51.06%)	16 (34.04%)	5 (10.64%)	2 (4.26%)	47			
Gender	Female	319 (34.84%)	364 (39.78%)	204 (22.28%)	29 (3.17%)	916	17.38	<0.001	3
	Male	268 (45.42%)	189 (32.03%)	116 (19.66%)	17 (2.88%)	590			
Year of study	1st Year	145 (31.42%)	209 (45.23%)	103 (22.29%)	5 (1.08%)	462	743.7	<0.001	9
	2nd Year	151 (43.39%)	100 (28.74%)	86 (24.71%)	11 (3.16%)	348			
	3rd Year	104 (29.89%)	150 (43.10%)	80 (22.99%)	14 (4.02%)	348			
	4th Year	187 (53.74%)	94 (27.01%)	51 (14.66%)	16 (4.60%)	348			

The impact of family history on the prevalence and severity of myopia was statistically significant, with a chi-squared value of 103.07 and a p-value of 0, indicating strong evidence of a familial influence on myopia. Individuals without a family history of myopia showed a lower prevalence rate of 616 (53.75%), whereas those with a family history exhibited a significantly higher prevalence rate of 300 (83.57%). Among the students with a family history of myopia, 322 (26.74%) had moderate myopia and 50 (4.18%) had high myopia (Tables 3, 4).

**Table 3:** Distribution of myopia severity across right and left eyes and family history of myopia

Variables	Category	Frequency	Percentage
Right eye	Emmetropia	587	38.98%
	Low myopia	553	36.72%
	Moderate myopia	320	21.25%
	High myopia	46	3.05%
Left eye	Emmetropia	590	39.18%
	Low myopia	564	37.45%
	Moderate myopia	309	20.52%
	High myopia	43	2.86%
Family history of myopia	No	1,147	76.16%
	Yes	359	23.84%

**Table 4:** Association between myopia severity and family history Pearson's chi-squared test; p < 0.001: Highly significant; p < 0.05: Significant

Family history of myopia	Emmetropia (n, %)	Low myopia (n, %)	Moderate myopia (n, %)	High myopia (n, %)	Total	Chi-squared value	p-value	Degree of freedom
No	531 (46.32%)	375 (32.69%)	213 (18.57%)	28 (2.44%)	1147	103.07	0	3
Yes	59 (16.43%)	189 (52.64%)	96 (26.74%)	15 (4.18%)	359			

A significant correlation was found between the age at which medical students developed myopia and its severity (χ² = 248.03, p < 0.001). Students who developed myopia early, between the ages of 5 and 10 years, exhibited more severe conditions, with 58 (51.33%) categorized as moderate and 25 (22.12%) as high myopia. Conversely, those who developed myopia during early adolescence (11-15 years) displayed a prevalence predominantly split between low and moderate myopia, while for those diagnosed from age 16, the condition was predominantly mild. These data underscores the need for early screening and potential interventions among younger populations to mitigate myopia progression (Tables 5, 6).

**Table 5:** Age of onset of myopia among study participants

Age of onset of myopia (years)	Frequency (n)	Percentage (%)
5 to 10 years	113	12.19%
11 to 15 years	462	49.84%
16 to present age	352	37.97%
Grand total	927	100.00%

**Table 6:** Association between myopia severity and age of onset Pearson's chi-squared test; p < 0.001: Highly significant; p < 0.05: Significant

Age of onset of myopia (years)	Emmetropia (n, %)	Low myopia (n, %)	Moderate myopia (n, %)	High myopia (n, %)	Total	Chi-squared value	p-value	Degree of freedom
5 to 10 years	0 (0.00%)	30 (26.55%)	58 (51.33%)	25 (22.12%)	113	248.03	0	6
11 to 15 years	1 (0.22%)	221 (47.84%)	221 (47.84%)	19 (4.11%)	462			
16 to present age	7 (1.99%)	302 (85.80%)	41 (11.65%)	2 (0.57%)	352			
Grand total	8 (0.86%)	553 (59.65%)	320 (34.52%)	46 (4.96%)	927			

When considering lifestyle factors with the corresponding chi-squared test results for near work (χ² = 15.14, p = 0.08), outdoor activity (χ² = 2.76, p = 0.97), screen time (χ² = 12.09, p = 0.2), and sleep time (χ² = 4.4, p = 0.88), it was found that the highest prevalence of near work exposure was among individuals spending three to six hours per day (355, 60.27%), while the lowest was in those engaging for only zero to one hour (1, 4.55%). One to three hours of screen time was the most common (239, 60.97%), whereas no participants reported more than six hours. Outdoor activity was strikingly low, with zero to one hour being the most prevalent (231, 60.16%), and no individuals exceeded six hours. Sleep patterns followed a moderate trend, with the majority sleeping five to eight hours (393, 61.90%), while 63 students (4.18%) reported sleeping under five hours (Table 7).

**Table 7:** Association of near work, outdoor activity, screen time, and sleep duration with myopia severity Pearson's chi-squared test; p < 0.001: Highly significant; p < 0.05: Significant

Variable	Category	Emmetropia (n, %)	Low myopia (n, %)	Moderate myopia (n, %)	High myopia (n, %)	Total	Chi-squared value	p-value	Degree of freedom
Near work activity	0 to 1 hour	1 (4.55%)	14 (63.64%)	6 (27.27%)	1 (4.55%)	22	15.14	0.08	9
	1 to 3 hours	4 (2.01%)	125 (62.81%)	59 (29.65%)	11 (5.53%)	199			
	3 to 6 hours	2 (0.34%)	355 (60.27%)	204 (34.63%)	28 (4.75%)	589			
	> 6 hours	1 (0.85%)	59 (50.43%)	51 (43.59%)	6 (5.13%)	117			
Outdoor activity	0 to 1 hour	4 (1.04%)	231 (60.16%)	127 (33.07%)	22 (5.73%)	384	2.76	0.97	9
	1 to 3 hours	4 (0.95%)	249 (59.14%)	149 (35.39%)	19 (4.51%)	421			
	3 to 6 hours	0 (0.00%)	69 (59.48%)	42 (36.21%)	5 (4.31%)	116			
	> 6 hours	0 (0.00%)	4 (66.67%)	2 (33.33%)	0 (0.00%)	6			
Screen time	0 to 1 hour	2 (3.17%)	33 (52.38%)	25 (39.68%)	3 (4.76%)	63	12.09	0.2	9
	1 to 3 hours	5 (1.28%)	239 (60.97%)	129 (32.91%)	19 (4.85%)	392			
	3 to 6 hours	1 (0.22%)	269 (60.31%)	155 (34.77%)	21 (4.71%)	446			
	> 6 hours	0 (0.00%)	12 (46.15%)	11 (42.31%)	3 (11.54%)	26			
Sleep time	0 to 5 hours	0 (0.00%)	39 (61.90%)	22 (34.92%)	2 (3.17%)	63	4.4	0.88	9
	5 to 8 hours	7 (1.09%)	393 (60.93%)	214 (33.21%)	31 (4.81%)	645			
	> 8 hours	1 (0.46%)	121 (55.25%)	84 (38.36%)	13 (5.94%)	219			

The average reading speed had the highest prevalence (329, 59.42%). Reading comprehension was strongest in those with a "Good" rating (275, 58.91%) and weakest among those classified as "Very Poor" (1.00%). Visual discomfort was most commonly reported as "Sometimes" (248, 60.63%), while only (2, 2%) individuals reported never experiencing discomfort.

The impact of myopia on daily activities was minimal for 156 low myopes (69.96%) but severe for six high myopes (50%), indicating a substantial burden at advanced stages. Lastly, the psychological burden of myopia was significantly associated with its severity (χ² = 28.26, p < 0.05). The psychological burden of myopia was absent in 217 patients with low myopia (64.38%); however 25 (56.82%) still reported a significant burden. Students with high myopia experience greater psychological distress, likely due to visual limitations and dependency on corrective measures (Table 8).

**Table 8:** Impact of myopia on reading speed, reading comprehension, visual discomfort, and psychological burden Pearson's chi-squared test; p < 0.001: Highly significant; p < 0.05: Significant

Variable	Category	Emmetropia (n, %)	Low myopia (n, %)	Moderate myopia (n, %)	High myopia (n, %)	Total	Chi-squared value	p-value	Degree of freedom
Reading speed	Average	5 (0.90%)	329 (59.42%)	195 (35.18%)	25 (4.52%)	554	20.2	0.06	12
	Fast	1 (0.35%)	170 (59.65%)	102 (35.79%)	12 (4.21%)	285			
	Slow	2 (4.00%)	26 (52.00%)	15 (30.00%)	7 (14.00%)	50			
	Very Fast	0 (0.00%)	19 (76.00%)	5 (20.00%)	1 (4.00%)	25			
	Very Slow	0 (0.00%)	9 (69.23%)	3 (23.08%)	1 (7.69%)	13			
Reading comprehension	Average	4 (1.01%)	240 (60.61%)	127 (32.08%)	25 (6.31%)	396	6.79	0.87	12
	Good	4 (0.86%)	275 (58.91%)	168 (36.00%)	20 (4.28%)	467			
	Poor	0 (0.00%)	9 (60.00%)	5 (33.33%)	1 (6.67%)	15			
	Very Good	0 (0.00%)	28 (59.57%)	19 (40.43%)	0 (0.00%)	47			
	Very Poor	0 (0.00%)	1 (50.00%)	1 (50.00%)	0 (0.00%)	2			
Visual discomfort	Always	0 (0.00%)	8 (42.11%)	9 (47.37%)	2 (10.53%)	19	7.98	0.78	12
	Never	2 (2.00%)	61 (61.00%)	34 (34.00%)	3 (3.00%)	100			
	Often	0 (0.00%)	60 (58.82%)	35 (34.31%)	7 (6.86%)	102			
	Rarely	2 (0.67%)	176 (59.26%)	106 (35.68%)	13 (4.38%)	297			
	Sometimes	4 (0.98%)	248 (60.63%)	136 (33.26%)	21 (5.13%)	409			
Effect of myopia on dialy activities	Extremely	0 (0.00%)	5 (41.67%)	6 (50.00%)	1 (8.33%)	12	41.21	<0.001	12
	Moderately	2 (0.82%)	129 (53.09%)	95 (39.09%)	17 (6.99%)	243			
	Not at All	5 (2.24%)	156 (69.96%)	52 (23.32%)	10 (4.48%)	223			
	Significantly	1 (1.19%)	35 (41.67%)	42 (50.00%)	6 (7.14%)	84			
	Slightly	0 (0.00%)	228 (62.48%)	125 (34.25%)	12 (3.29%)	365			
Psychological burden of myopia	Extremely	0 (0.00%)	2 (25.00%)	5 (62.50%)	1 (12.50%)	8	28.26	<0.05	12
	Moderately	1 (0.53%)	98 (52.13%)	72 (38.30%)	17 (9.04%)	188			
	Not at all	3 (0.89%)	217 (64.38%)	105 (31.16%)	12 (3.56%)	337			
	Significantly	0 (0.00%)	25 (56.82%)	13 (29.55%)	6 (13.64%)	44			
	Slightly	4 (1.14%)	211 (60.29%)	125 (35.71%)	10 (2.86%)	350			

## Discussion

It has been observed that myopia has a high prevalence of 947 (61.55%) among Andhra Pradesh medical students; thus, it can be considered a relevant issue in this student population, which is significantly higher than the global average among university students (27.17%) as reported by Grigoraș et al. [6]. The prevalence was also significantly higher in female students (597, 65.19%) than in male students (322, 54.58%), which is consistent with the findings of studies in European and Asian populations [7,8]. In addition, our results indicated that students with a family history of myopia had a 300 (83.57%) prevalence rate, consistent with earlier reports indicating a strong genetic factor. A previous study found 332 (66.39%) positive family history and with studies showing that people with both parents having refractive errors are twice as likely to develop myopia (OR = 2.01, 95% CI (1.2, 3.4)) [9,10].

The investigation also found an important correlation between early age of myopia onset (5-10 years) and greater severity levels (p < 0.05), underlining the importance of early screening and treatment [11,12]. Lifestyle factors like near-work time, screen time, and outdoor activity, though displaying trends related to myopia severity, were not significant statistically (p = 0.08, 0.2, 0.97, respectively). However, previous studies suggest that these factors may influence the progression of myopia over time. Those with moderate to severe myopia (p < 0.05) scored higher on distress and reliance on correction, especially in clinical situations, and affected their confidence and everyday functioning. Research has also shown a 0.9D rise in myopia progression during COVID-19 caused by increased screen exposure [13,14]. 

Different studies have suggested that the association between myopia, family history, and lifestyle factors varies among different populations. In Rawalpindi, Pakistan, it was observed that 61.8% of medical students became myopic in adulthood and 23.6% were myopic from childhood [15]. In Gujarat, 48.3% of medical students had refractive errors and the most prevalent was myopia (90% of cases) [9]. On the other hand, a Saudi Arabian study of medical students reported a prevalence rate of 57.9%, and studies conducted in Norway and Denmark reported rates of 50.3% and 50%, respectively. A higher prevalence of 61.55% was observed in our study, perhaps because of varying academic workloads, lifestyle habits, and access to eye care [7].

The contribution of screen use and near work to the development of myopia remains controversial. In a study conducted in Rawalpindi, Pakistan, no effect of screen time or study time on myopia was found, which is consistent with our results (p = 0.2) [15]. Conversely, a Taiwanese study found that high academic performance was associated with higher myopia prevalence among children [16]. However, another study determined that 26% of myopic students experienced high study pressure, and 8% experienced very high study pressure, indicating that academic load was a causative factor. In addition, excellence in academics was prevalent among myopic students, who scored 70-90% for 52% and above 90% for 30% [17].

A meta-analysis of 61,946 adults in Europe reported a combined myopia incidence of 24.2%, with a considerably higher prevalence among young adults (47.2% between the ages of 25 and 29). The average prevalence in Poland was 24.1%, while an Argentine study noted that myopia accelerated due to COVID-19, with a refractive change of -0.9D during quarantine, in contrast to -0.3D per annum before the pandemic [14]. Although our research did not directly evaluate pandemic-related factors, the trend of myopia progression with higher screen time merits further study.

Our results confirm those of earlier research illustrating the powerful role of family history in myopia development. According to one study, students with one myopic parent had a 35% chance of becoming myopic, and those with two myopic parents had an 85% chance [16]. Similarly, 65% of myopic students reported having siblings with myopia, which was much higher than 23% among non-myopic students [7]. Our study also observed an even greater familial impact (83.57%), which again validates the genetic predisposition hypothesis among Indian medical students.

These findings are corroborated by studies that have shown a high prevalence of myopia among medical students. Other studies, such as the ones by and Grigoraș et al. and Chalasani et al., have replicated the same myopia prevalence pattern among young adults [6,18]. In addition, Bibi et al. and Oszczędłowski et al. also supported the heredity of myopia by presenting a high dependency of myopia severity on family history, which is associated with the nature of the disorder [12].

The prevalence of myopia in our study (61.55%) is in agreement with that of Oszczędłowski et al., who reported a prevalence of 52.97% among Eastern Polish medical students [12]. Yet, in European studies like Grigoraș et al.'s, it was found that Romanian medical students presented a higher prevalence of 73.8% [6]. Conversely, Chinese studies have found significantly higher rates of prevalence, including Shi et al. (92.52%) and Wang et al. (70.50%), indicating that regional and environmental conditions are key factors [19-21].

Our study is differs in some respects. Whereas Grigoraș et al. reported an increasing prevalence of myopia with increasing years of study, we observed the highest prevalence among first-year students and a decrease in later years [6]. This discrepancy may be due to differences in academic pressures, study patterns, or self-selection biases in different settings. First-year students can experience an abrupt increase in near-work activities and screen time because of academic changes, while senior students can adapt coping mechanisms, such as improved study habits or more effective use of corrective lenses. Other reasons, such as differential access to vision care, greater awareness, and proactive interventions in advanced academic years, can explain this declining trend [21].

Strengths and limitations

One of the key strengths of this study is its large sample size of 1,506 students from several medical colleges, which increases the generalizability of the results. This study design also permitted a thorough examination of the demographic and lifestyle determinants of myopia. The use of validated questionnaires provided a thorough evaluation of the prevalence, severity, and risk factors of myopia. Another strength is the inclusion of psychological impact assessments, which highlights the wider implications of myopia beyond vision loss.

However, this study also has some limitations. The use of self-reported information may have resulted in recall bias, especially for lifestyle factors such as screen time and near work. Moreover, the cross-sectional design constrains our ability to infer causality between myopia and its risk factors. Second, the study did not include measurements of axial length and cycloplegic refraction, which are more objective estimates of myopia development. Additionally, cognitive bias may have influenced responses since participants, as medical students, possess prior knowledge of myopia and its risk factors. Future studies should incorporate clinical validation methods, such as cycloplegic refraction or vision screening camps, to cross-verify self-reported refractive error data and enhance reliability. Prospective studies involving longitudinal follow-up and objective measures of refraction would further strengthen these results.

Implications

Thus, the results of this study emphasize the importance of early detection programs for myopia among medical students, particularly those with a family history of myopia. In addition, adopting measures such as vision breaks at regular intervals, optimal light conditions, and publicity campaigns on optimal eye care regimens may minimize the effects of myopia. Since there is a substantial psychological load from high myopia, mental health services and counseling also need to be included as myopia management components. Healthy visual habits must also be addressed at the institutional policy level through, promoting outdoor play, ergonomically designing classrooms, and facilitating regular vision screenings. Promoting the use of techniques such as the 20-20-20 rule (gazing away from screens every 20 minutes for 20 seconds at a distance of 20 feet) and correct study lighting could potentially reduce strain-related myopia progression. In addition, future research should investigate specific interventions such as digital eye strain management and blue light filter use to ascertain their potential in mitigating myopia burden.

## Conclusions

A high prevalence of myopia among medical students in Andhra Pradesh was observed in this study, showing links between myopia and lifestyle factors such as, continued near-work activities, excessive screen time, and reduced outdoor exposure. A higher risk is observed in female students especially in their first year of medical school. This study underlines the need for early detection through regular eye check-ups and adoption of lifestyle adjustments, to slow the progression of myopia. Furthermore, advocating for targeted vision care intervention needs, this study emphasize the negative impact of myopia on academic performance, daily life, and psychological well-being. The study results help us understand the prevalence of myopia in academic settings and provide a basis for research and initiatives for prevention.

## Figures and Tables

**Figure 1:**
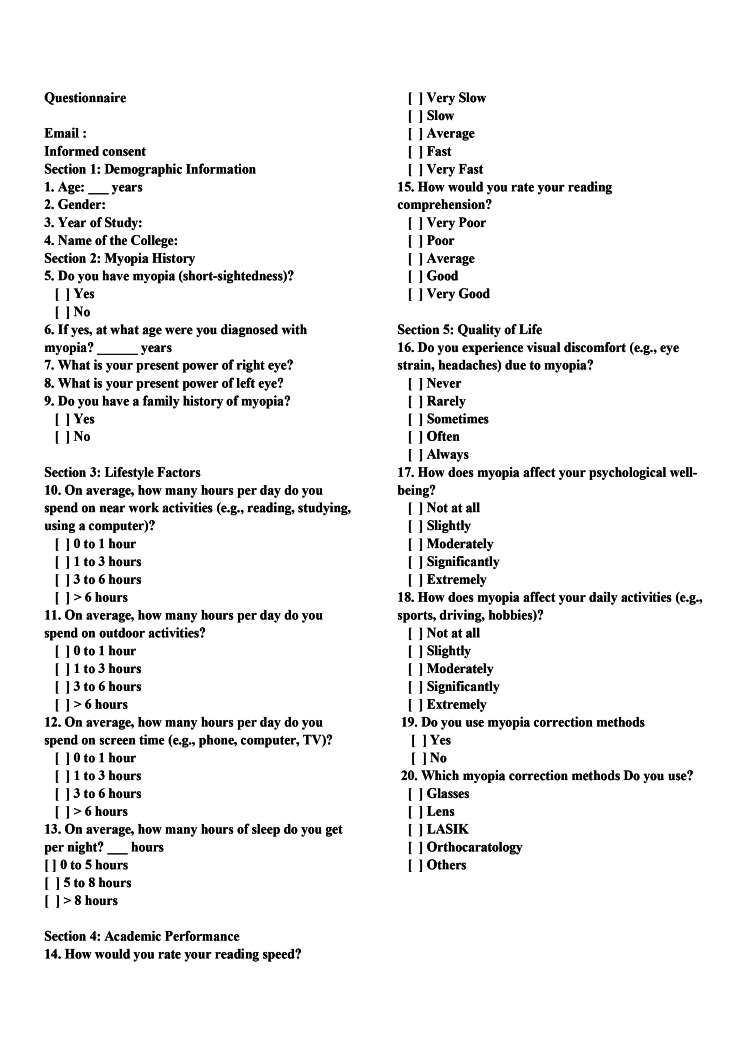
Questionnaire
